# Suitability of virtual plaster models superimposed with the lateral cephalogram for guided paramedian orthodontic mini-implant placement with regard to the bone support

**DOI:** 10.1007/s00056-020-00238-2

**Published:** 2020-07-06

**Authors:** Stephan Christian Möhlhenrich, Maximilian Brandt, Kristian Kniha, Anna Bock, Andreas Prescher, Frank Hölzle, Ali Modabber, Golamreza Danesh

**Affiliations:** 1 Department of Orthodontics, University of Witten/Herdecke, Alfred-Herrhausen-Str. 45, 58455 Witten, Germany; 2 Private Practice for Orthodontics, Blumenstraße 29, 73728 Esslingen, Germany; 3 Department of Oral and Maxillofacial Surgery, University Hospital of Aachen, Pauwelsstraße 30, 52074 Aachen, Germany; 4 Institute of Molecular and Cellular Anatomy, Medical Faculty, RWTH-Aachen, Pauwelsstraße 30, 52074 Aachen, Germany

**Keywords:** Template, Guided surgery, Oral implants, Dimensional measurement accuracy, Orthodontic anchorage procedures, Bohrschablone, Schablonengeführte Chirurgie, Orale Implantate, Dimensionale Messungen, Kieferorthopädische Verankerungsverfahren

## Abstract

**Purpose:**

The purpose of this study was twofold: first, to evaluate the precision of guided orthodontic mini-implant (OMI) placement planned on virtual superimposition of plaster models and lateral cephalograms with regard to the bone support and, second, to investigate the effects of silicone guide extension.

**Methods:**

A total of 40 OMIs were placed in the paramedian area of the anterior palates of 20 cadaver heads. Digitalized models and the corresponding lateral cephalograms were superimposed for planning the OMI positions, and tooth-supported (TS) and soft-tissue-supported (STS) templates were manufactured. Thereafter, postoperative cone beam computed tomography (CBCT) was performed, and the straight (A) and right-angle distance (B) from the implant tip to the nasal floor, the distance from the implant shoulder to the hard palate (C) and the angle (α) between the implant and palate plane with the preoperative (T0) and postoperative (T1) positions were measured.

**Results:**

The postoperative distances A, B, and C were less than the planned implant positions. However, significant difference between T0 and T1 was only noted in terms of distance A using the TS templates (T0: 4.7 ± 2.3 mm, T1: 3.0 ± 2.3 mm; *p* = 0.008) and distance B using the STS template (T0: 3.1 ± 3.5 mm, T1: 2.3 ± 3.2 mm; *p* = 0.041). There were no significant differences in all average deviations (∆ Ceph/CBCT) between the two templates.

**Conclusions:**

Guided OMI placement planned by virtual superimposition of digitized models and the corresponding lateral cephalogram is fundamentally feasible. However, the position closer to the nasal floor needs critical assessment for correct implantation. The silicone template expansion seems to have only a minor effect on transfer accuracy.

## Abstract (de)

**Zweck:**

Die Studie verfolgte 2 Ziele: Zum einen wurde die Übertragungspräzision einer schablonengeführten Insertion kieferorthopädischer Mini-Implantate (OMI) basierend auf einer virtuellen Planung von überlagerten Situationsmodellen und korrespondierenden FRS(Fernröntgenseiten)-Aufnahmen unter Berücksichtigung des Knochenangebots bewertet, zum anderen wurde der Einfluss der Ausdehnung der Bohrschablone untersucht.

**Methoden:**

Insgesamt wurden 40 OMIs paramedian in den anterioren Gaumen von 20 Körperspendern inseriert. Zuvor waren digitalisierte Modelle und korrespondierende FRS-Aufnahmen miteinander überlagert und darauf basierend zahn- (ZGS) und schleimhautgetragene Schablonen (SGS) für die Insertion hergestellt worden. Nach der Insertion erfolgte eine digitale Volumentomographie (DVT). Anschließend wurden der direkte Abstand (A) sowie der rechtwinklige Abstand (B) zum Nasenboden sowie der Abstand der Implantatschulter zum Gaumen (C) und der Winkel zwischen Implantat und Gaumen (α) in der präoperativen Planung (T0) und der postoperativen Implantatposition (T1) gemessen.

**Ergebnisse:**

Die postoperativen Abstände A, B und C im DVT waren geringer als in der FRS-basierten Planung. Jedoch wurden signifikante Unterschiede zwischen T0 und T1 nur für Abstand A nur nach Verwendung der ZGS (T0: 4,7 ± 2,3 mm, T1: 3,0 ± 2,3 mm; *p* = 0,008) und für Abstand B unter zur Hilfenahme der SGS (T0: 3,1 ± 3,5 mm, T1: 2,3 ± 3,2 mm; *p* = 0,041) festgestellt. Hinsichtlich der Übertragungsgenauigkeit (∆ FRS/DVT) gab es keine signifikanten Unterschiede zwischen beiden Schablonen.

**Schlussfolgerungen:**

Die schablonengeführte OMI-Insertion basierend auf der virtuellen Überlagerung digitalisierter Modelle und korrespondierender FRS-Aufnahmen ist grundsätzlich möglich, allerdings der kürzere Abstand zum Nasenboden verlangt eine entsprechende Berücksichtigung bei der Planung. Die Ausdehnung der Bohrschablone hat nur einen geringen Effekt auf die Übertragungsgenauigkeit.

## Introduction

Orthodontic mini-implants (OMI) for skeletal anchorage are being increasingly employed in routine clinical practice because of their relatively easy insertion and removal along with their relative inexpensiveness [2]. Additionally, the need for patient compliance is largely reduced with OMI, and borderline cases can easily undergo orthodontic treatment without surgery [1, 16, 25]. Proximity to the adjacent teeth serves as a major risk factor for OMI failure. Kuroda et al. observed a success rate of 35.3% in root contacts following lower jaw implementation [15]. In addition, other studies concluded that root contact is associated with reduced mini-implant success rates [6, 15, 22]. However, Kadioglu et al. contended that the root surfaces in contact with OMIs rapidly and almost totally repair after the removal of the implant or the orthodontic force [11].

In particular, the anterior palate is often used for placement due to high blood supply to the bone in this region, which facilitates high stability of the implant and reduces the risk of injuries to the adjacent structures [21]. These risks include trauma to the dental roots, nerve involvement, perforation into the nasal or maxillary sinus, chronic persistent sinus inflammation, and anchorage loss [14]. The anterior hard palate has been well investigated as a region for skeletal anchorage in the field of orthodontics. This, in particular, includes three-dimensional (3D) computed tomography studies of the bone volume and quality [4, 8, 9, 12, 13, 18, 24].

Several studies have already reported the use of drilling guides for OMI placement [7, 17, 19, 23]. Recently, Cassetta et al. and De Gabriele et al. described new CAD/CAM surgical templates that were specifically designed for palatal orthodontic appliances to minimise implant site preparation and OMI placement [5, 7]. Following the fusion of cone beam computed tomography (CBCT) and dental digital model images using dedicated software, the drilling guide was created on the resulting 3D images and subsequently manufactured using a 3D printer.

In this context, Wilmes et al. demonstrated the possibility of superimposition with lateral cephalograms [27]. Cassetta et al. performed postoperative CBCT after OMI placement and compared results with preoperative planning. They reported that this template permitted controlled and accurate palatal OMI insertions in all dimensions. However, this means that the patient would be exposed to additional radiation during CBCT. Given this fact, Maino et al. introduced two cases of OMI insertion using drilling templates which were planned to be inserted on virtual models superimposed on lateral cephalograms [19]. They determined that these templates helped avoid injuries to the anatomical structures and reduced patient discomfort. Moreover, the authors concluded that the reduced costs and radiation exposure are additional benefits.

Jung et al., for the first time, reported the use of lateral cephalograms for vertical analysis of bone density and height in the anterior palate [10]. They measured vertical bone height on lateral radiographs as well as CBCT images and found a comparable vertical bone dimension in the median and parasagittal planes using both techniques. Furthermore, they reported that vertical bone density, as displayed on lateral cephalometry, reflects the minimum bone height rather than the maximum bone height in the median plane.

Braces can lead to various problems during the insertion of rigid, tooth-supported (TS) drilling templates. Therefore, in a previous investigation, the authors of this study investigated transfer accuracy of OMI placement at the anterior palate using silicone-based guides with different expansions [20]. According to Maino et al., planning was performed on virtual plaster models superimposed with the corresponding lateral cephalograms. Following placement, transfer accuracy was evaluated on postoperative intraoral scans supported by scan bodies to determine the final OMI position relative to the preoperative planning models using automatic surface registration based on an iterative closest point algorithm. Sufficient control of OMI placement similar to the CAD/CAM templates was noted. Although the planned OMI position was slightly less accurate than the guided dental implantology, it seemed to be sufficient for receiving an orthodontic appliance.

However, these deviations may lead to injuries to the adjacent structures, particularly perforations of the nasal floor. Therefore, the purpose of this study was twofold: first, to retrospectively analyze the previously published cadaveric data to evaluate the precision of fully guided OMI placement planned by superimposition of virtual plaster models and the corresponding lateral cephalograms with regard to the real bone density and, second, to investigate the effects of silicone guide expansion on possible deviations.

## Materials and methods

The Institute of Molecular and Cellular Anatomy of the University Hospital RWTH Aachen, Germany, approved this study. The Ethics Committee of the Medical Faculty of the RWTH Aachen (EK 219/16) recommended that only the intuitional approval of the Molecular and Cellular Anatomy of the University Hospital of the RWTH Aachen, which was given in the present investigation is required for cadaveric studies. Two OMIs were inserted in the paramedian area of the anterior palates of 20 non-fixed fresh heads of body donors, who donated their bodies for research and education (14 men and 6 women; mean [range] age 71 [66–83] years). These human cadaver heads belonged to a body donor group from a previously published investigation, and this study represents an ongoing evaluation [20]. The placement was performed using TS (*n* = 10, implants *n* = 20) and soft-tissue-supported (STS; *n* = 10, implants *n* = 20) templates. Maximum loss of four teeth, not more than two per side, was considered as the inclusion criterion of this study.

### Template fabrication and implantation

**Fig. 1:**
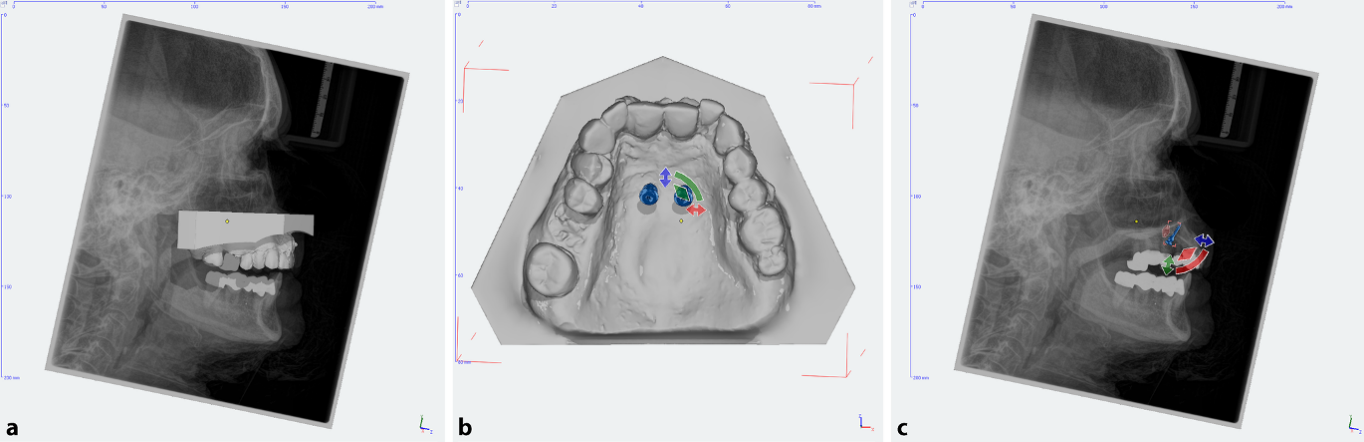
**a** Preoperative lateral cephalogram superimposed with the corresponding virtual plaster model. **b** Appropriate paramedian position of the orthodontic mini-implants with an interimplant distance of 8 mm. **c** Lateral cephalogram with a virtually positioned implant at an inclination of 70–80° to the occlusal plane for fine adjustment **a** Präoperative Fernröntgenseitenaufnahme mit entsprechendem überlagertem virtuellem Gipsmodell. **b** Paramedian positionierte kieferorthopädische Mini-Implantate mit einem interimplantären Abstand von 8 mm. **c** Fernröntgenseitenaufnahme mit virtuell positionierten Implantaten in einem Winkel von 70–80° zur Okklusionsebene zur Feineistellung

For virtual planning, Impregum Penta (3M ESPE, Neuss, Germany) was used to create plaster models of the upper jaw based on impressions. Furthermore, the corresponding lateral cephalograms (Orthophos SL 2D, Dentsply Sirona, York, PA, USA) were created. After pouring the impressions with super-hard plaster (Alpenrock, Amann Girrbach, Koblach, Austria), the placement models were digitized using a 3D model scanner (orthoX scan, Dentaurum, Ispringen, Germany), and lateral cephalograms along with the corresponding models were superimposed using dedicated software (TAD match, OnyxCeph, Image Instruments GmbH, Chemnitz, Germany; Fig. 1). The implant position was determined on the virtual plaster model, and fine adjustment and inclination angle were set on a lateral radiograph. All OMIs (OrthoLox, Promedia Medizintechnik A Ahnfeldt GmbH, Siegen, Germany) measured 2 × 10 mm^2^. The distance between implants was 8 mm, the angle of insertions was set about 70–80° to the occlusal plane and the height of the mini-implant heads was slightly above the resistance center of the molars. Finally, the working models were manufactured using a 3D printer (Form 2, Formlabs, Somerville, MA, USA). In addition, templates were produced using the same 3D printer.

**Fig. 2:**
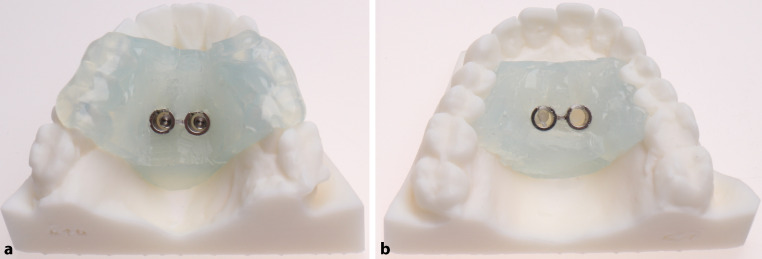
Three-dimensional (3D)-printed working models with tooth-supported (**a**) and soft-tissue-supported (**b**) templates Dreidimensional (3-D) gedruckte Arbeitsmodelle mit zahn- (**a**) und schleimhautgetragener Schablone (**b**)

To create the TS and STS templates, a drill sleeve was precisely placed on the previously virtually planned mini-implant pillars and was surrounded with two-component silicone (Transpasil, KANIEDENTA GmbH & Co. KG, Herford, Germany) (Fig. 2).

After manufacturing, the surgical templates were transferred to the human heads and positioned on the teeth and/or soft tissues. Thereafter, OMIs were inserted without predrilling using a contra-angle handpiece drive (Prosthodontic implant driver, W&H, Bürmoos, Austria). The placement automatically stopped after OMI reached the final depth, separating the implant from the insertion aid.

### Accuracy measurements

**Fig. 3:**
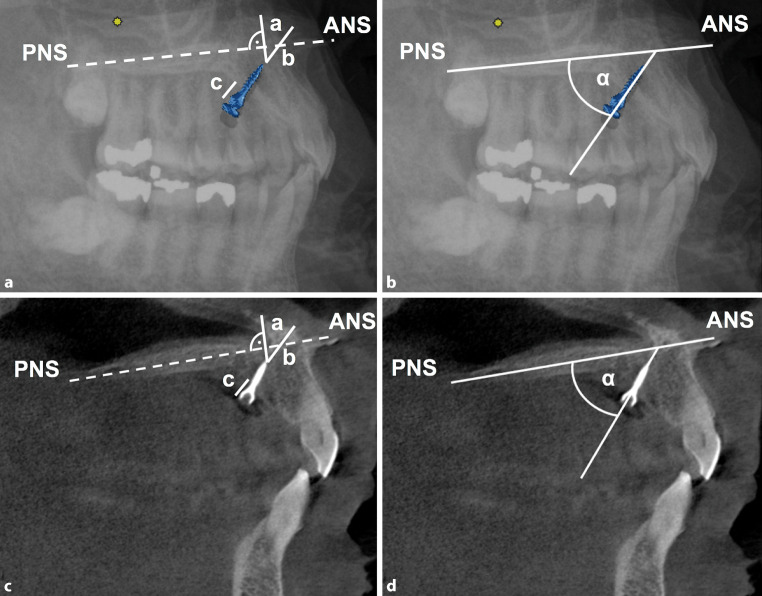
Radiographic measurement on lateral cephalogram (**a**, **b**) and the corresponding CBCT scans (**c**, **d**). *Line a* distance from the implant tip to the nasal floor at the right angle to the hard palate; *line b* straight distance from the implant tip to the nasal floor; *line c* posterior distance from the implant shoulder to the anterior hard palate and angle α: angle between the implant and palate plane (ANS-PNS) Radiologische Messungen in der Fernröntgenseitenaufnahme (**a**, **b**) und den korrespondierenden DVT (digitale Volumentomographie)-Aufnahmen (**c**, **d**). *Linie a* Abstand von der Implantatspitze zum Nasenboden im rechten Winkel zum harten Gaumen; *Linie b* gerader Abstand von der Implantatspitze zum Nasenboden; *Linie c* hinterer Abstand von der Implantatschulter zum harten Gaumen, Winkel α: Winkel zwischen Implantat und Gaumenebene (ANS-PNS)

Postoperative CBCT scans (Galileos, Sirona, Bensheim, Germany) were obtained. Linear and angular measurements were performed on lateral cephalograms of the virtually positioned OMIs (*n* = 10) as well as on CBCT scans in the corresponding parasagittal planes (*n* = 20; Fig. 3). The palate plane (distance between the anterior nasal spine [ANS] and posterior nasal spine [PNS]) served as the reference line:

- Line A: distance from the implant tip to the nasal floor at a right angle to the hard palate
- Line B: straight distance from the implant tip to the nasal floor
- Line C: posterior distance from the implant shoulder to the anterior hard palate
- Angle α: angle between the implant and palate planes (ANS-PNS)

### Statistical analysis

All measurements were repeated after a 2-week interval by the same investigator. Calibration of the investigator was assessed with the intraclass correlation coefficient (ICC). ICC was >0.85 for all variables, with the overall ICC ranging between 0.92 and 0.98. The D’Agostino and Pearson test was used to test data normality distribution. Wilcoxon matched-pairs signed rank test was employed for comparing preoperative planned and postoperative achieved implant positions, and Mann–Whitney test was employed to analyze differences in average deviation depending on the surgical template. The level of significance was set at *P* ≤ 0.05 using the statistical program Prism (version 7, GraphPad Software Inc., La Jolla, CA, USA). All results are expressed as the mean ± standard deviation values.

## Results

**Fig. 4:**
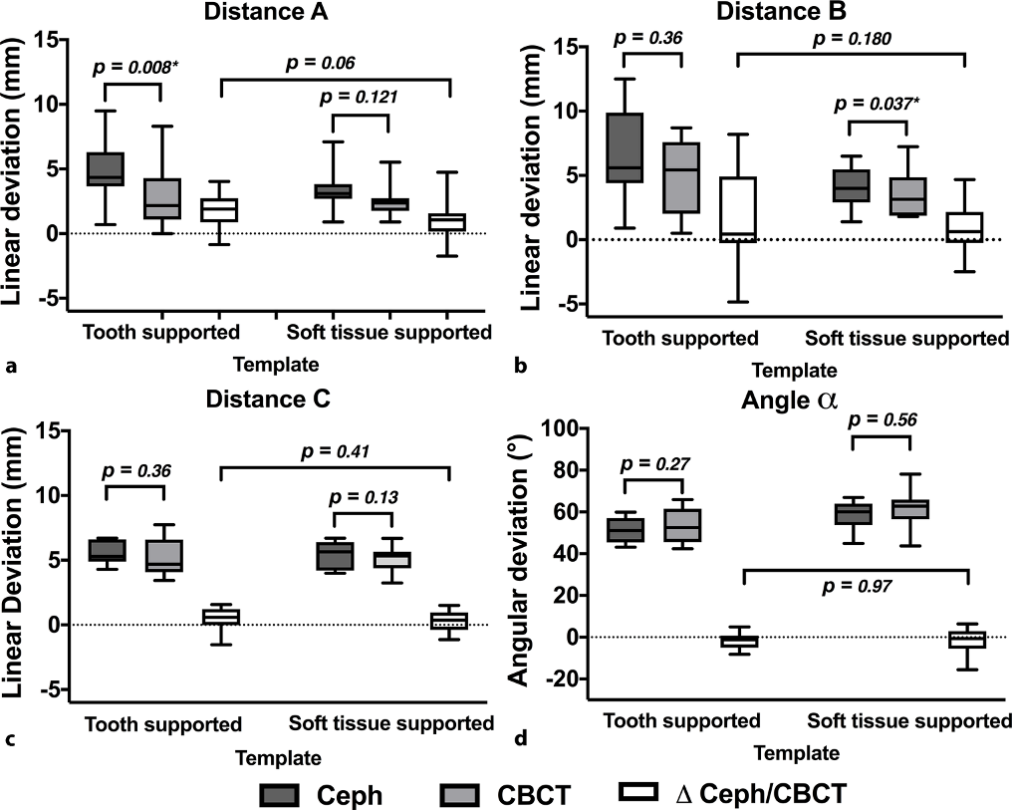
Boxplots of the linear and angular measurements depending on the surgical template (*TS* tooth-supported, and *STS* soft-tissue-supported) and the corresponding *p*-values of the statistical comparisons: **a** distance A, **b** distance B, **c** distance C, **d** angle α Boxplots der linearen und angulären Messungen in Abhängigkeit von der chirurgischen Bohrschablone (*TS* zahngetragen, *STS* schleimhautgetragen) mit den entsprechenden *p*-Werten der statistischen Vergleiche: **a** Abstand A,** b **Abstand B,** c** Abstand C,** d** Winkel α

The outcomes of linear and angular measurements depending on the surgical template (TS and STS) are presented in Table 1. The corresponding boxplots and *p*-values of statistical comparisons (cephalograms vs. CBCT scans and ∆Ceph/CBCT: TS vs. STS) are presented in Fig. 4.

**Table 1:** Radiological determined distances A–C (mm) and angle α (°) between the orthodontic mini-implant (OMI) and palatal plane (ANS-PNS) after planning paramedian OMI insertion (lateral cephalogram, Ceph) and OMI placement (cone beam computed-tomography, CBCT) using tooth supported (TS) or soft tissue supported (STS) templates Radiologisch ermittelte Abstände A, B und C (mm) sowie Winkel α (°) zwischen kieferorthopädischem Miniimplantat (OMI) und Gaumenebene (ANS-PNS) in der virtuellen Planung der paramedianen OMI-Insertion (Fernröntgenseitenaufnahme, FRS) und nach der OMI-Insertion (digitale Volumentomographie, DVT) unter Verwendung von zahn- (TS) oder schleimhautgetragenen Schablonen (STS)

Measurement	Template	Imaging	*N*	Mean	SD	Min	Max
*Distance A*	TS	Ceph	10	4.7	2.3	0.7	9.5
		CBCT	20	3.0	2.3	0.0	8.3
		∆ Ceph/CBCT	20	1.7	1.2	−0.9	4.0
	STS	Ceph	10	2.3	3.1	−2.7	7.1
		CBCT	20	1.6	2.1	−2.4	5.5
		∆ Ceph/CBCT	20	1.6	1.5	0.2	5.7
*Distance B*	TS	Ceph	10	6.8	3.8	0.9	12.5
		CBCT	20	4.9	2.9	0.5	8.7
		∆ Ceph/CBCT	20	1.8	3.6	−4.8	8.2
	STS	Ceph	10	3.1	3.5	−2.9	6.5
		CBCT	20	2.3	3.2	−3.2	7.2
		∆ Ceph/CBCT	20	1.8	1.9	0.1	8.5
*Distance C*	TS	Ceph	10	5.6	0.9	4.3	6.7
		CBCT	20	5.2	1.5	3.4	8.7
		∆ Ceph/CBCT	20	0.5	1.0	−2.5	1.6
	STS	Ceph	10	5.4	1.1	4.0	6.7
		CBCT	20	5.2	1.0	3.2	6.7
		∆ Ceph/CBCT	20	0.7	0.4	0.2	1.5
*Angle α*	TS	Ceph	10	51.3	5.8	43.1	59.9
		CBCT	20	53.2	8.0	42.4	65.9
		∆ Ceph/CBCT	20	3.3	2.7	0.2	8.2
	STS	Ceph	10	58.8	7.1	44.9	66.9
		CBCT	20	61.4	10.3	43.7	84.1
		∆ Ceph/CBCT	20	4.8	5.7	−5.0	21.6

*SD* standard deviation

Mean distance between the implant tip and nasal ground was less with the use of STS templates for distances A and B. There were significant differences between measurements on lateral cephalograms and CBCT in terms of distance A alone for the TS templates (*p* = 0.008) and for distance B alone for the STS template (*p* = 0.041). Furthermore, ∆Ceph/CBCT was small and negative for both the templates with regard to linear measurements, indicating a closer final position of the implant tip to the nasal floor as planned preoperatively. However, there were no significant differences in average deviations (∆Ceph/CBCT) between the two templates in terms of distances A, B, and C. Regard angular deviations, there were no significant differences between the preoperative planning and postoperative implant positions as well as between the two templates.

## Discussion

In an earlier investigation, the authors of this study found that the use of silicone surgical guides provides sufficient control of OMI placement and that these guides are comparable to the CAD/CAM templates [20]. However, compared to that with the known guides used in dental implantology, OMI position is less accurate with these guides. Nevertheless, these appear to be sufficient for loading an orthodontic appliance. However, only the oral situation was focused upon and no information was provided regarding the implant deviation relative to the surrounding bone or adjacent anatomical structures in that study. Therefore, the present study represents a continuation of the said previous investigation. The primary objective of this study was to evaluate accuracy of the achieved OMI position compared to the virtually planned position based on the digitalized models superimposed with the corresponding lateral cephalograms. The secondary objective of this study was to investigate the effects of extension of silicone surgical template with regard to the finally achieved OMI position.

The use of surgical guides based on virtual planning of OMI placement in the anterior palate can be suitable in patients with low vertical bone height, cleft palate, or palatally displaced teeth. Possible benefits of its application include the protection of anatomical structures and achievement of the best possible skeletal anchorage.

Becker et al. demonstrated that the insertion angle and OMI position are important factors which must be taken into account in OMI placement planning to achieve the best possible bone support [3]. They also reported that an optimal insertion positions seems to be at the height of the first and second premolars in the anterior palate. Furthermore, posterior tipping was beneficial at anterior positions, while anterior tipping was advantageous at posterior positions.

Various drilling templates have been described for the placement of palatal orthodontic implants. De Gabriele et al. superimposed a plaster cast on a CBCT image to identify optimal sites for mini-implant placement in the anterior hard palate [7]. In this context, Wilmes et al. reported that superimposition with lateral cephalometric radiographs is also possible [27]. After virtual placement planning, a surgical insertion guide, called Easy Driver, was produced by rapid prototyping, which allowed for insertion through the guide using a special contra-angle screwdriver. Hereby, the placement of the mini-implants as well as of the orthodontic appliances can be achieved in the oral cavity during the same appointment. Cassetta et al. also introduced a CAD/CAM surgical guide based on 3D images created by the fusion of dental digital model images and CBCT scans using dedicated software [5]. Likewise, Tonsun et al. presented a surgical guide that contained metal drill housing based on the plaster cast sections with tracing of a lateral cephalogram of the anterior maxilla [26] Subsequently, Maino et al. presented two cases of OMI insertion in which a superimposition of virtual plaster models and lateral radiographs was used for manufacturing drilling templates [19]. Along with a reduction in costs and radiation exposure, they reported possible improvements of the predictability of the final implant position and increase in patient comfort. Maino et al. used 3D-printed surgical guides [19], and Möhlhenrich et al. used the templates manufactured by a two-component silicone gripped around the stainless steel drilling sleeves that were positioned on the pillars in a mini-implant position on a working model [20].

Jung et al. have proposed the ability to use lateral cephalograms for evaluating the vertical bone support for palatal insertion of OMIs [10]. In a cadaveric study, they investigated the vertical palatal bone dimensions on lateral cephalometry and CBCT with regard to the implications for palatal implant placement. Lateral radiographs and CBCT scans of 18 human skulls were obtained, and the nasal floor and the oral hard palate of all skulls were lined for lateral cephalometry with a tin foil for contrast enhancement. Afterwards, the quantity of vertical bone in CBCT was analyzed, as measured on lateral radiographs, in the median and parasagittal planes and at a minimum bone height. A markedly higher median palatal bone height was noted on CBCT than on the lateral cephalograms. In addition, the strongest association between both radiological imaging techniques was observed at the minimum palatal bone height. Therefore, the authors concluded that lateral cephalograms could provide an accurate and adequate assessment of vertical bone before the paramedian insertion of palatal implants. Specifically, the vertical bone supply, as measured on the lateral radiographs, reflects the minimum bone height rather than the maximum bone height in the median plane; therefore, preoperative CT or CBCT seems to be only indicated when the lateral cephalometry reveals marginal quantity of bone.

For the first time, this study analyzed the accuracy of OMI placement based on simplified planning by superimposition of virtual plaster models and the corresponding lateral cephalograms with regard to the final position related to the bone support. Although a previous investigation detected sufficient precision for receiving an orthodontic appliance regarding the OMI position within the oral cavity [20], the measured deviations may still be sufficiently large to affect the implant position in the bone to an extent that perforations of the nasal floor are possible.

In the present investigation, the distances A, B and C were less than the planned implant positions, indicating that the finally achieved position was closer to the nasal floor. A statistically significant reduction was found for the TS templates for distance A and for the STS templates for distance B. Deviations for distance C were comparatively smaller. This may be due to angular changes of the implant within the bone, which cannot be considered in the two-dimensional view. However, no insertion perforated the nasal floor. No benefit was found between the two template types (TS vs. STS) during the comparison of average deviation of the three distances (∆Ceph/CBCT). Nevertheless, the linear aberrations can be viewed as critical. The average deviation was ~1.7 ± 1.2 mm, with a maximum value up to 4.0 mm, for distance A and ~1.6 ± 1.5 mm, with a maximum value up to 5.7 mm, for distance B. These large fluctuations in values should be taken into account when selecting the mini-implant length.

Regarding the angle between implant and palate plane (ANS-PNS), only a slightly but not statistically significant increase was noted between the preoperative planning and postoperative clinical implant positions as well as between the two drilling templates. However, it must be noted that this measurement was performed on CBCT in the parasagittal plane of the respective mini-implant. As a result, an anatomical change of the reference line (palate plane, ANS-PNS) is conceivable; however, the validity of this measurement is limited.

Comparing the results of this study with those of previously published studies, the vertical deviations were less. In a previous study, the vertical deviations for the TS and STS templates were ~2.34 ± 0.74 and ~2.14 mm ± 0.73, respectively [20]. Our observations corroborate the findings of Jung et al. that the vertical bone dimension, as displayed on lateral cephalometry, reflects the minimum bone height rather than the maximum bone height in the median plane [10]. Finally, there is a concern that the fabrication method of the insertion guides in the present study does not comply with common techniques. Perhaps, these printed templates are more suitable to eliminate errors due to manipulations by a technician. Further studies should investigate this limitation.

## Conclusions

Guided mini-implant insertion based on virtual planning by superimposition of digitized plaster models and the corresponding lateral cephalogram is fundamentally feasible. Within the limits of this cadaveric study, the deviations between the planned and achieved OMI positions mainly accounted for more linear deviations than angular ones. The linear deviations with regard to possible perforation of the nasal floor must be critically assessed. A safety distance of 2 mm is recommended when selecting the implant length. Expansion of the silicone template seems to have only a minor effect on transfer accuracy.
